# St. Bartholomew's Hospital

**Published:** 1909-05-15

**Authors:** 


### ST. BARTHOLOMEW'S HOSPITAL.

### OPENING OF THE NEW PATHOLOGICAL BLOCK.

The Lord Mayor, on Friday, May 7, opened the new pathological block at St. Bartholomew's Hospital, a description of which will shortly appear in The Hospital with accompanying plans. The Lord Mayor, who was accompanied by Miss Nancy Truscott and the Sheriffs, was received on arrival at the hospital by Lord Sandhurst (the treasurer), the almoners, and the senior members of the medical and surgical staff, with Mr. T. Hayes (the clerk). After inspecting the new building the party proceeded to the great hall, where a large company had assembled. Lord Sandhurst read an address to the Lord Mayor signed by himself on behalf of the governors, which dealt with the long and intimate historical associations which have existed between successive Lord Mayors and Corporations of the City of London and the hospital of St. Bartholomew since before the time of Sir Thomas Gresham; and pointed out the advantages to the community which must follow the opening of this new wing. The Lord Mayor, having received the key from Mr. E. B. I'Anson, the architect, declared the new building open. He said the opening of the pathological block would mean an era of increased usefulness of the hospital. The great advances in curative and preventive medicine during the last twenty years have been largely due to the study of pathology. He hoped that the studies carried on in the new building would lead to some discovery concerning the treatment of cancer and tuberculosis. Assisted by the City guilds and the merchant princes of London, the Corporation had often helped St. Bartholomew's in the past; and the Lord Mayor expressed his intention of calling the attention of the guilds, the merchants, and the public generally, as well as of the Corporation, to the present financial necessity of the hospital, arising out of the desire of the governing body that the institution should be brought up to date, and made what it ought to be—one of the best hospitals in the world. The Prince of Wales, the President of the hospital, had assured him of his keen interest in the institution and in the present state of its finances.

Sir Rubert Boyce, who was sent by the Colonial Office to the West Indies to study tropical sanitation, has written a letter home in which, according to the *Times*, he states that immense progress has been made since 1905 in regard to tropical sanitation and to mosquito-borne diseases. Now all the health authorities prosecute and inflict heavy fines for having larvæ of mosquitoes on premises or in houses. The teachings of tropical medicine have taken root and have brought about a revolution in tropical sanitation, and, above all, every one sees now how practicable it all is and what a return they get for any small action they may have made. In another ten years diseases like yellow fever and malaria will be things of the past in islands like the West Indies and in many other parts of the tropical world. If it had not been for the impetus which the Liverpool School of Tropical Medicine in the West Indies has given to the study of these diseases, we could not have accomplished what has been done.

At the quarterly court of the directors of the Society for Relief of Widows and Orphans of Medical Men, held last month under the chairmanship of Dr. Blandford, President, it was reported that since the last meeting three members of the court of directors had died: Mr. T. Laurence Read, Vice-President, and Dr. Eastes and Dr. Chas. Baker, and votes of condolence with their families were passed. Membership of the Society is open to any registered medical practitioner who at the time of his election is resident within a twenty-mile radius of Charing Cross. The subscription is two guineas per annum, and the Society confines its assistance to the widows and orphans of deceased members. The offices of the Society are at 11 Chandos Street, Cavendish Square, W., and information may be obtained from the Secretary at that address. The invested funds of the Society now amount to over £100,000. The annual general meeting will be held on Thursday, May 20.

A festival dinner in aid of the funds of the City of London Hospital for Diseases of the Chest was held last week at the Trocadero Restaurant. The Lord Mayor, who was accompanied by the Sheriffs, presided, and in proposing "Prosperity to the Hospital," said that since its foundation upwards of 41,000 people had visited it as patients, and that 750,000 out-patients had received treatment. Last year about 1,150 cases were treated in the hospital, while 57,000 out-patients were dealt with. Every bed in the hospital was now occupied for the first time in its history. Mr. H. H. Nelson, responding, urged that money given to charities should not be liable to the payment of income-tax. He put that forward as something which the Government might reasonably consider. Mr. Alderman and Sheriff Hanson proposed "The Medical Staff," and Dr. Heron responded. The secretary announced donations and subscriptions amounting to upwards of £2,300.

Year by year the circulation of *Printers' Pie* has grown, and by the sale of the new issue, which appeared on May 12, Mr. Spottiswoode hopes to be able to provide a larger contribution than before to the funds of the Printers' Pension Corporation, on whose behalf it is produced. This year's president of the corporation is the Prince of Wales. In addition to the Printers' Pension Corporation, the Booksellers' Provident Institution, the Newsvendors' Benevolent and Provident Institution, the Newspaper Press Fund, and the Artists' General Benevolent Institution will benefit by the sale of the annual. The following authors and artists, among others, have helped in the production: The Duke of Argyll, G. B. Burgin, Egerton Castle, Lieutenant-Colonel Newnham-Davis, Austin Dobson, Tom Gallon, Keble Howard, W. S. Maugham, Baroness Orczy, Barry Pain, Mostyn T. Pigott, William Le Queux, Frank Richardson, W. Pett Ridge, Adrian Ross, Owen Seaman, and George R. Sims. There are also pictures by Cecil Aldin, G. D. Armour, H. M. Bateman, Lewis Baumer, George Belcher, H. M. Brock, Tom Browne, Rene Bull, Dudley Hardy, W. K. Haselden, John Hassall, L. Raven Hill, George Morrow, Will Owen, F. Pegram, E. L. Stampa, Lance Thackeray, Thorpe, F. H. Townsend, Lawson Wood, and Starr Wood. The price of *Printers' Pie, 1909*, is 1s., and it is published by the Sphere and Tatler, Ltd.

### FOOD CONGRESS AT PARIS, 1909.

The successful Congress in connection with the suppression of frauds in food held last year at Geneva, will be succeeded by a similar Congress to be held in Paris during October of the present year. The Congress is under the auspices of the Society of the White Cross of Geneva, presided over by Mr. C. H. Vuille of that city, and it is contemplated that the forthcoming Congress will, numerically, far exceed that which was held at Geneva, when about 400 delegates, representing 29 different nations, were present. At the Paris Congress, the principal object will be to define methods to prevent the fraudulent adulteration of food, but there will also be sections devoted to chemical products, pharmaceutical preparations, mineral waters, and similar substances. The list of foods includes all the various well-known domestic products. There will be two principal sections—technical and industrial—and those two sections will endeavour to define clearly what purity of the various substances discussed really means. It is obvious, also, that the combination of technical and scientific men along with actual producers is likely to lead to clear and practical definitions. It is anticipated that there will be a considerable representation of British interests in connection with the technology and science of the subject, as well as in connection with agriculture and manufactures of food products. Those who intend to participate in the Congress, either by the appointing of delegates or as individuals, should apply for information to Mr. Loudon M. Douglas, College of Agriculture, Edinburgh. The General Secretary is Mr. Robert Fazy, 42 Rue du Rhone, Geneva.

